# Correction to “Comparison of multi‐subject ICA methods for analysis of fMRI data”

**DOI:** 10.1002/hbm.70006

**Published:** 2024-08-25

Erhardt, E.B. , Rachakonda, S. , Bedrick, E.J. , Allen, E.A. , Adali, T. , & Calhoun, V.D. (2011). Comparison of multi‐subject ICA methods for analysis of fMRI data. Human Brain Mapping, 32, 2075–2095. 10.1002/hbm.21170 21162045 PMC3117074

In the author affiliations section, the affiliation of Tülay Adali was incorrect. It was stated as “Department of CSEE, University of Maryland, Baltimore, Maryland.” This should have read: “Department of CSEE, University of Maryland Baltimore County, Maryland.”

We apologize for this error.

